# Effects of intact and hydrolysed blue whiting proteins on blood pressure and markers of kidney function in obese Zucker fa/fa rats

**DOI:** 10.1007/s00394-020-02262-9

**Published:** 2020-05-14

**Authors:** Aslaug Drotningsvik, Åge Oterhals, Svein Are Mjøs, Linn Anja Vikøren, Ola Flesland, Oddrun Anita Gudbrandsen

**Affiliations:** 1 Dietary Protein Research Group, Department of Clinical Medicine, University of Bergen, Haukeland University Hospital, 5021 Bergen, Norway; 2 TripleNine Vedde AS, 6030 Langevåg, Norway; 3 Nofima, 5828 Bergen, Norway; 4 Department of Chemistry, University of Bergen, 5020 Bergen, Norway; 5 Department of Clinical Science, University of Bergen, 5021 Bergen, Norway

**Keywords:** Fish proteins, Blood pressure, Hypertension, Obesity, Zucker, Rat

## Abstract

**Purpose:**

To investigate the effects of diets containing intact or hydrolysed proteins from blue whiting (*Micromesistius poutassou*) on the development of high blood pressure and markers of kidney function in obese Zucker fa/fa rats which are prone to develop hypertension and renal failure.

**Methods:**

Male rats were fed isocaloric diets containing either intact blue whiting whole meal (BW-WM), blue whiting protein hydrolysate prepared with Alcalase^®^ (BW-HA) or blue whiting protein hydrolysate prepared with Protamex^®^ (BW-HP) as 1/3 of total protein with the remaining 2/3 as casein, or casein as sole protein source (control group). Blood pressure was measured at Day 0 and Day 32. Rats were housed in metabolic cages for 24 h for collection of urine in week 4. After 5 weeks, rats were euthanized and blood was drawn from the heart. The renin and angiotensin-converting enzyme (ACE) inhibition capacities for casein and blue whiting proteins were measured in vitro.

**Results:**

The blood pressure increase was lower in rats fed diets containing blue whiting proteins when compared to the control group, whereas markers of kidney function were similar between all groups. The three blue whiting proteins inhibited renin activity in vitro, whereas casein had no effect. The in vitro ACE inhibition was similar for casein, BW-WM and BW-HP proteins, whereas BW-HA protein was less potent.

**Conclusion:**

Blue whiting protein feeding attenuated the blood pressure increase in obese Zucker fa/fa rats, possibly mediated through the renin–angiotensin system and without affecting markers of kidney function.

**Electronic supplementary material:**

The online version of this article (10.1007/s00394-020-02262-9) contains supplementary material, which is available to authorized users.

## Introduction

High blood pressure is associated with increased risk of chronic renal and cardiovascular diseases [1–3]. Lifestyle modifications are recommended to prevent hypertension [4], and several studies show that a high fish intake is associated with lower blood pressure [5–12]. A blood pressure-lowering effect of fish may be mediated through the angiotensin–renin system, since peptides with angiotensin-converting enzyme (ACE) inhibiting capacities in vitro have been identified in fish fillet, skin and backbone [13], but evidence concerning in vivo effects of fish proteins on blood pressure is limited. Angiotensinogen is cleaved by renin to the biologically inactive angiotensin I, which is then converted to the active vasoconstrictor angiotensin II by ACE, with the cleavage of angiotensinogen by renin as the rate determining step [14].

The obese Zucker fa/fa rat is a much used model of genetic obesity and has been shown to be well suited for studies on metabolic complications and treatments of obesity and hypertension [15]. These rats develop visible obesity from the age of 3–4 weeks and develop an age-related increase in blood pressure already before the age of 10 weeks [15–17], and with increasing age they also spontaneously develop proteinuria and focal segmental glomerulosclerosis leading to renal failure [18].

Fish meals (non-hydrolysed) produced from Atlantic cod (*Gadus morhua*) residual materials or fillet have shown potential to prevent blood pressure increase and beneficially affect markers of kidney function in obese Zucker fa/fa rats [19, 20]. Blue whiting (*Micromesistius poutassou*) is a small pelagic fish primarily used to produce fish meal for aquaculture feed and belongs to the family Gadidae included in the order Gadiformes (codfishes) together with, among others, the Atlantic cod. The market for blue whiting as whole fish or fillet for human intake is limited by factors such as small size and discoloration [21], and blue whiting products based on fish meal or protein hydrolysates may be one option to improve the utilization of this fish species. We have recently shown that a water-soluble protein meal from blue whiting has a hypocholesterolemic effect in obese Zucker fa/fa rats [22], but more knowledge on the potential health effects of proteins from blue whiting is warranted to expand its usability in new products for human consumption. Enzymatic hydrolysis enables efficient recovery of proteins from fish and fish by-products and produces protein fractions with higher content of small peptides that may exert effects as bioactive compounds. Blue whiting protein hydrolysates have been shown to have ACE-inhibiting properties in vitro [23, 24], but the effects of blue whiting hydrolysates on blood pressure and markers of kidney function in vivo have not yet been investigated.

The primary objective of the present study was to compare the effects of diets containing proteins from headed and gutted blue whiting as whole meal or protein hydrolysates on the development of high blood pressure in obese Zucker fa/fa rats. The secondary objectives were to investigate any changes in markers of kidney function, organ damage, inflammation and oxidative stress, to examine the in vitro renin and ACE-inhibiting properties of the blue whiting proteins, and to explore the possible impact of dietary components in the blue whiting protein meals that could affect blood pressure development. Our hypothesis was that blue whiting protein intake would attenuate the development of high blood pressure in obese Zucker fa/fa rats possibly through inhibition of the renin–angiotensin system, and beneficially affect markers of kidney function.

## Methods

### Ethical statement

The study protocol was approved by the National Animal Research Authority (Norway) in accordance with the Animal Welfare Act and the Regulation of animal experiments (Approval No. 2014/6979). All applicable international, national and institutional guidelines for the care and use of animals were followed.

### Animals and diets

Twenty-four male obese Zucker fa/fa rats (HsdHlr:ZUCKER-Leprfa) were obtained from Harlan Laboratories (Indianapolis, IN, USA). The rats were housed in pairs in Macrolon IV cages (EHRET GmbH & Co.) in a room with a 12 h light/dark cycle, at 20–23 °C and a relative humidity of 55–65%. Rats were acclimatized for a minimum of 7 days under these conditions, before being randomly allocated to intervention groups or control group, with six rats in each group. The intervention period started when the rats were 8–9 weeks old and weighed 319 ± 11 g. The number of rats per group was chosen based on previous experience from studies on the development of high blood pressure in obese Zucker fa/fa rats [25]. Rats were fed modified semi-purified diets based on the American Institute of Nutrition’s recommendation for growing laboratory rodents (AIN-93G) [26] with the addition of 1.6 g methionine/kg diet as recommended by Reeves [27], and differed only in their protein sources (Table 1). All diets contained 20wt% protein. The AIN-93G diet was used instead of the AIN-93 M diet for maintenance containing 15 wt% protein, since rats would be in the growth phase throughout the intervention period (based on growth charts for Zucker rats from Harlan Laboratories, https://www.envigo.com). In addition, obese Zucker rats have an impaired protein metabolism, which leads to inferior protein utilization and requires a greater protein intake to maintain a maximal rate of protein gain during growth [28]. The three intervention diets contained fish proteins produced from the same batch of headed and gutted blue whiting. All blue whiting protein diets contained 1/3 (by weight) of total dietary protein from blue whiting and the remaining 2/3 (by weight) of protein was casein. Blue whiting proteins were in the form of either blue whiting whole meal (BW-WM), blue whiting protein meal hydrolysate prepared with Alcalase^®^ (BW-HA), or blue whiting protein meal hydrolysate prepared with Protamex^®^ (BW-HP). Casein was the sole protein source in the control diet (Table 1). All ingredients were purchased from Dyets Inc. (Bethlehem, PA, USA) except casein which was purchased from Sigma-Aldrich (Munich, Germany) and fish proteins which were prepared from blue whiting by Nofima (Bergen, Norway).

**Table 1:** Composition of the experimental diets

Contents (g/kg diet)	Control diet	BW-WM diet	BW-HA diet	BW-HP diet
Casein^a^	216.0	144.0	144.0	144.0
Blue whiting whole meal protein^b^	–	93.3	–	–
Blue whiting protein hydrolysed with Alcalase^®c^	–	–	87.7	–
Blue whiting protein hydrolysed with Protamex^®d^	–	–	–	85.3
Cornstarch	511.7	490.5	496.3	498.5
Sucrose	90.0	90.0	90.0	90.0
Cellulose	50.0	50.0	50.0	50.0
Soybean oil	70.0	70.0	70.0	70.0
t-Butylhydroquinone (TBHQ)	0.015	0.015	0.015	0.015
Mineral mix (AIN-93-MX)	35.0	35.0	35.0	35.0
Vitamin mix (AIN-93-VX)	10.0	10.0	10.0	10.0
l-Methionine	1.6	1.6	1.6	1.6
l-Cystine	3.0	3.0	3.0	3.0
Choline bitartrate^e^	2.5	2.5	2.5	2.5
Growth and maintenance supplement^f^	10.0	10.0	10.0	10.0

*BW-WM* blue whiting whole meal *BW-HA* blue whiting protein hydrolysate prepared with Alcalase^®^, *BW-HP* blue whiting protein hydrolysate prepared with Protamex^®^

^a^Contains 92.5% crude protein, < 1% fat, 8% moisture, < 1% ash

^b^Contains 71.4% crude protein, 5% fat, 8% moisture, 9% ash

^c^Contains 76.0% crude protein, < 0.01% fat, 5% moisture, 13% ash

^d^Contains 78.2% crude protein, < 0.01% fat, 3% moisture, 12% ash

^e^Contains 41% choline

^f^Contains vitamin B12 (40 mg/kg) and vitamin K1 (25 mg/kg) mixed with sucrose (995 g/kg) and dextrose (5 g/kg)

### Preparation of blue whiting proteins

The blue whiting was frozen on-board the fishing vessel, landed and then partially thawed at ambient temperature, and headed and gutted before further freeze storage until use. To produce whole meal, the frozen headed and gutted fish was partially thawed overnight, added equal amount of water and heated to 90 °C in a 150 L steam-heated kitchen cooker. After 10 min holding time, the cooked material was mechanically dewatered in a P13-SCR double-screw press (Stord Bartz AS, Bergen, Norway). The press liquid was heated to 90 °C and run through a Jesma VS 20/65 Roto-Fluid sieve (Jesma, Velje, Denmark; 100 μm sieve net opening) to remove suspended solids. The Jesma solids was mixed with the press cake and dried to a press cake fish meal on a TG1 fluid bed dryer (Retsch GmbH & Co. KG, Germany) at 70 °C. The Jesma liquid was evaporated on a four-stage falling film evaporator (APV Anhydro, Søborg, Denmark) at 60–100 °C. The concentrate was mixed with an equivalent amount of press cake fish meal and dried on a TG1 fluid bed dryer at 70 °C to obtain a whole meal. The whole meal was milled on a Retsch ZM-1 centrifugal mill (Retsch GmbH, Haan, Germany) with a ring sieve aperture of 0.75 mm.

Two different protein hydrolysates were produced from the headed and gutted blue whiting. The headed and gutted fish was partially thawed and processed on a meat grinder with 7.5 mm aperture and was added an equal amount of water in a 200 L stirred tank reactor. The fish slurry was heated to 50 °C under continuous stirring before addition of the following combinations of enzyme (given on crude protein basis) and residence time: Alcalase^®^ 2.4 L (Novozymes AS, Bagsværd, Denmark) 0.5%, residence time 60 min and Protamex^®^ (Novozymes AS, Bagsværd, Denmark) 1%, residence time 60 min. After the predefined residence time, the hydrolysates were heated to 90 °C and kept at this temperature level for 10 min to inactivate the enzyme. The hydrolysates were filtrated on a Jesma VS 20/65 Roto-Fluid sieve (Jesma, Velje, Denmark; 100 μm sieve net opening) before microfiltration by use of Membralox (Pall Corporation, Portsmouth, UK) ceramic membranes with pore size 100 nm, removing all fats in the hydrolysates. The permeates were concentrated in a four-stage falling film evaporator (APV Anhydro, Soeborg, Denmark) at 60–100 °C before final drying. The Alcalase^®^ protein hydrolysate was dried in a Christ Gamma 1–16 LSC freeze dryer (Martin Christ Gefriertrocknungsanlagen GmbH, Osterode am Harz, Germany), and the Protamex^®^ protein hydrolysate in a Niro P-6.3 spray drier (Niro, Sjøborg, Denmark) with inlet and outlet temperature 200 and 94 °C, respectively. The dried Alcalase^®^ protein hydrolysate was milled on a Retsch ZM-1 centrifugal mill with a ring sieve aperture of 1.0 mm, while the Protamex^®^ protein hydrolysate was used as is.

### Design

Rats were fed ad libitum for 5 weeks, with free access to tap water and chewing sticks. Rats were weighed weekly during the intervention period. One week before end point, rats were housed individually in metabolic cages (Ancare Corp., NY, USA) for 24 h for collection of urine and measurement of feed intake, without fasting in advance. Blood pressure was measured in conscious rats at baseline (Day 0) and 3 days before end point (Day 32). At the end of the experimental period, after a 12 h fast, rats were euthanized while anaesthetized with isoflurane (Isoba vet, Intervet, Schering-Plough Animal Health, Boxmeer, The Netherlands) mixed with nitrous oxide and oxygen. Blood was drawn from the heart and collected in Vacuette Z Serum Clot Activator Tubes for isolation of serum (Greiner Bio-One, Austria) and in Vacuette K2EDTA tubes (Greiner Bio-One) for isolation of plasma. Liver and epididymal white adipose tissue (WATepi) were dissected out and frozen. All biological samples were stored at − 80 °C.

### Analyses of diets

Contents of amino acids (except α-aminobutyric acid, β-alanine, γ-aminobutyric acid, citrulline, 4-hydroxyproline, 1-methylhystidine and 3-methylhistidine), energy and sodium content in diets, and crude protein, fat, moisture, ash and peptide size distribution of blue whiting protein meals, were measured by Nofima BioLab (Bergen, Norway). Amino acids were measured using HPLC [29]. Dietary caloric content was determined by a bomb calorimeter method in accordance with ISO9831:1998 [30] using a Parr 6400 calorimeter (Parr Instrument Company, Illinois). Dietary sodium content was determined by flame atomic absorption spectrometry in accordance with ISO6869:2000 [31] using Perkin Elmer Analyst 400 with an AS 90plus autosampler (PerkinElmer, Massachusetts). Crude protein was determined according to the Kjeldahl method [32]. Fat content was determined gravimetrically after chloroform/methanol extraction [33]. Moisture content was measured gravimetrically after drying in a forced-air oven at 103 ± 1 °C for 4.5 h [34]. Total ash content was determined gravimetrically after incineration at 550 °C [35]. Peptide size distributions for the blue whiting proteins were measured by HPLC size exclusion chromatography as described previously [36]. Fatty acid composition of diets was analysed by gas chromatography after lipid extraction as described below.

α-Aminobutyric acid, β-alanine, γ-aminobutyric acid, citrulline, 4-hydroxyproline, 1-methylhystidine (π-methylhistidine), 3-methylhistidine (τ-methylhistidine) and taurine were quantified in diets as described below, after total acid hydrolysis (6 M HCl, 24 h, 110 °C).

### Renin and ACE inhibition in vitro

Casein and BW-WM protein were added Trizma buffer (50 mM, pH 8.0) and hydrolysed using trypsin from bovine pancreas (T1426 from Sigma) at 45 °C for 4 h as recommended by Shalaby et al. [37]. The two blue whiting protein hydrolysates (BW-HA, BW-HP) were not hydrolysed with trypsin prior to analyses. Protein in hydrolysates were quantified on the Cobas c111 system (Roche Diagnostics GmbH, Mannheim, Germany) using the TP2 kit from Roche. Renin inhibition was measured using the Renin Assay Kit (MAK157, from Sigma-Aldrich) as described in the user manual. ACE-inhibition was measured using the method by Shalaby et al. [37], as previously described [20].

### Blood pressure measurements

Systolic and diastolic blood pressures were measured at baseline and end point. Rats were pre-warmed in a heating cabinet at 32 °C for 30 min before blood pressure was measured using the tail-cuff method (CODA-6, Kent Scientific Corporation, Torrington, CT, USA).

### Analyses in serum, plasma, urine and kidney

Serum concentrations of creatinine, alanine transaminase and aspartate transaminase (the latter two were measured with pyridoxal phosphate activation), and urine concentrations of creatinine, total protein, carbamide, uric acid and ammonium were analysed on the Cobas c111 system (Roche Diagnostics GmbH, Mannheim, Germany) using the CREP2 (Creatinine plus ver.2), ALTL (Alanine aminotransferase acc. IFCC), ASTL (Aspartate aminotransferase), TP2 (Total Protein Gen.2 monochromatic), UREAL (Urea/BUN), UA2 (Uric Acid ver.2) and NH3L (Ammonia) kits from Roche Diagnostics. Sodium concentrations in serum and urine were analysed on the Cobas c111 system (Roche Diagnostics GmbH, Mannheim, Germany) using the Ion-Selective Electrode module from Roche Diagnostics.

Plasma and urine concentrations of cystatin C were quantified using the Mouse/Rat Cystatin C Quantikine^®^ ELISA (catalogue number MSCTC0) from R&D Systems, Bio-Techne, MN.

Plasma for glutathione measurements was added to four volumes of ice-cold 5% meta-phosphoric acid, mixed and stored on ice for 15 min before centrifugation, and the supernatant was stored at − 80 °C until analysis. Total and oxidized glutathione were analysed using the Glutathione (GSSG/GSH) detection kit (ADI-900-160) from Enzo Life Sciences AG, Lausen, Switzerland. Reduced glutathione was calculated as the difference between total and oxidized glutathione.

Serum concentrations of monocyte chemoattractant protein (MCP)-1, interleukin (IL)-1b, IL-6 and tumor necrosis factor (TNF) α were measured using the MILLIPLEX^®^ MAP Rat Cytokine/Chemokine Magnetic Bead Panel (RECYTMAG-65 K) from EMD Millipore Corp. (St. Charles, MO).

Kidneys were homogenized in Tris-buffer (pH 7.8) before analyses of T cell immunoglobulin mucin-1 (TIM-1), using the Rat TIM-1/KIM-1/HAVCR Quantikine^®^ ELISA (catalogue number RKM100) from R&D Systems. Kidney protein content was measured with the Bradford dye-binding method [38] using Protein Assay Dye Reagent (Bio-Rad Laboratories, Germany) with bovine serum albumin (Bio-Rad Protein Assay Standard II, Bio-Rad Laboratories) as standard.

### Amino acids in plasma, urine and HCl-hydrolysed diets

Free amino acids in EDTA-plasma and urine, and total amino acids in HCl-hydrolysed diets were quantified by reverse-phase high performance liquid chromatography, using the S 433 Automatic Amino Acid Analyser (Sykam GmbH, Eresing, Germany), equipped with integrated dual-channel photometer for the detection of amino acids at 440–570 nm, cooled autosampler and reagent storage, and integrated vacuum degasser. The autosampler and reagent storage were kept at 13–14 °C, and total acquisition time was 111 min. Mobile phases were lithium citrate buffer A-1 (0.12 N, pH 2.90), lithium citrate buffer B-1 (0.30 N, pH 4.20), lithium citrate/borate buffer C-4 (0.30 N, pH 8.0) and regeneration solution (0.45 N) (all for physiological program, from Sykam GmbH), with post-column derivatization with ninhydrin (Sykam GmbH). The mobile phases were delivered according to the following scheme at constant flow of 0.450 ml/min: 0–12.50 min, 100% buffer A-1; 12.60–38.00 min: 74% buffer A-1/26% buffer B.1; 38.10–50.00 min: 46% buffer A-1/54% buffer B-1; 50.10–62.00 min: 22% buffer A-1/78% buffer B-1; 62.10–63.50 min: 100% buffer B-1; 63.60–71.00 min: 76% buffer B-1/24% buffer C-4; 71.10–85.00 min: 100% buffer C-4; 85.10–94.00 min: 80% buffer C-1/20% Regeneration solution, 94.10–107.00 min: 74% buffer C-4/26% regeneration buffer, 107.10–111.00 min: 100% regeneration buffer. Column oven temperature was 39 °C (0–88 min), 60 °C (88–89 min); 70 °C (89–111 min). Amino acids were derivatized post-column with ninhydrin (Sykam GmbH), with a constant reactor temperature of 130 °C. Clarity Amino chromatography station version 7.4.1.99 (Sykam GmbH) was used for data acquisition and analysis. Plasma and urine samples for quantification of amino acids were prepared as follows: four volumes of plasma or urine was added to one volume of 5-sulfosalicylic acid dihydrate (Sigma-Aldrich, Munich, Germany), containing the internal standard norleucine (Sigma-Aldrich), to precipitate proteins. After centrifugation (2000 × *g*, 5 min), the supernatant from plasma was diluted 1:1 with Lithium citrate buffer A-1 (Sykam GmbH), whereas the supernatant from urine was diluted 2:1 with the same buffer. The amino acid standard stock solution for physiological samples (PH, from Sykam GmbH) added to l-glutamine (Sigma-Aldrich) was used as calibrator. γ-Aminobutyric acid and β-alanine were quantified in urine but were not found in plasma, and tryptophan was quantified in plasma but could not be quantified in urine. Otherwise the same compounds were analysed in plasma and urine. Only α-aminobutyric acid, β-alanine, γ-aminobutyric acid, citrulline, 4-hydroxyproline, 1-methylhystidine (π-methylhistidine), 3-methylhistidine (τ-methylhistidine) and taurine were quantified using this method in the diets.

### Fatty acids in serum, liver, WATepi and diets

Lipids in liver and diets were extracted according to the method described by Bligh and Dyer [33] using a mixture of methanol and chloroform, before methylation. Serum and WATepi were methylated without prior extraction of lipids. Fatty acids in liver and diet extracts, serum and WATepi were analysed by gas chromatography as described previously [39].

### Statistical analyses

Statistical analyses were conducted using SPSS Statistics version 25 (SPSS, Inc., IBM Company, Armonk, NY, USA). All variables were evaluated for normality using the Shapiro–Wilks test, Q–Q plots and histograms and most variables were normally distributed. One-way analysis of variance (ANOVA) was used to compare groups, followed by the Fisher’s LSD post hoc test to determine significant differences between groups when appropriate. All biological data are presented as means ± standard deviations. Bivariate correlations were calculated using Pearson’s two-tailed test of significance. Results from the measurements of renin and ACE inhibition by dietary proteins are presented as means ± standard error of mean. The cut off value for statistical significance was set at a probability of 0.05. One rat in the control group was excluded from all statistical analyses due to apparent disease, thus results are presented as *N* = 5 rats for the control group and *N* = 6 rats for each of the intervention groups.

## Results

### Blue whiting protein peptide size distribution

Analyses of peptide size distribution showed that the enzymatic hydrolysis protocols were sufficient to produce > 80% of peptides with a molecular size below 4000 g/mol (83.5 and 91.2% in hydrolysates BW-HA and BW-HP, respectively) and the hydrolysates contained negligible quantities of large proteins > 20,000 g/mol (Table 2). The water-soluble protein fraction of the non-hydrolysed BW-WM consisted of 29.5% peptides with molecular weight > 20,000 g/mol and the peptide fraction with molecular weight < 200 g/mol (comprising free amino acids and miscellaneous water-soluble components absorbing light with a wavelength of 214 nm) amounted to 45.3%.

**Table 2:** Molecular weight distribution of water-soluble peptides

g/mol	BW-WM protein	BW-HA protein	BW-HP protein
> 20,000	29.5	0.4	< LOD
20,000–15,000	4.5	0.3	< LOD
15,000–10,000	5.1	1.2	0.4
10,000–8000	2.4	1.8	0.9
8000–6000	2.1	4.0	2.2
6000–4000	1.8	8.6	5.4
4000–2000	1.5	18.4	15.1
2000–1000	0.8	20.2	20.1
1000–500	0.7	14.9	19.3
500–200	6.4	12.2	17.5
< 200^a^	45.3	17.8	19.2

*BW-WM* blue whiting whole meal, *BW-HA* blue whiting protein hydrolysate prepared with Alcalase^®^, *BW-HP* blue whiting protein hydrolysate prepared with Protamex^®^, *LOD* level of detection

^a^The peptide fraction with molecular weight < 200 g/mol comprises free amino acids and miscellaneous water-soluble components absorbing light with a wavelength of 214 nm)

### Diets, dietary intake, growth and organ weights

Dietary contents of indispensable amino acids were in general similar between the diets (Table 3); however the content of the conditionally essential amino acid arginine was higher in all blue whiting protein diets. β-Alanine, 3-methylhistidine and taurine were detected only in blue whiting protein diets, with highest amount in BW-HA and BW-HP. 4-Hydroxyproline and 1-methylhystidine were also found only in diets containing blue whiting meal, with little differences between these diets. The content of γ-aminobutyric acid was lower in the blue whiting containing diets compared to the control diet. The amounts of α-aminobutyric acid and citrulline were below the level of detection in all diets. Arachidonic acid (20:4n-6) and the n-3 PUFAs 20:5n-3, 22:5n-3 and 22:6n-3 (Σn-3 PUFA 0.11 wt%) were detected only in the BW-WM diet, otherwise fatty acid composition was similar between diets.

**Table 3:** Dietary content of indispensable amino acids, non-proteogenic amino acids, arginine, taurine, and fatty acids

	Control diet	BW-WM diet	BW-HA diet	BW-HP diet
*Amino acids (g/kg diet)*				
Arginine	6.9	9.4	9.6	9.2
β-alanine^b^	ND	0.20	0.48	0.40
Citrulline^b^	ND	ND	ND	ND
γ-aminobutyric acid^b^	0.03	0.01	0.01	0.01
Histidine	5.6	5.2	5.2	4.9
4-Hydroxyproline^b^	ND	0.54	0.57	0.42
Isoleucine	10.2	9.8	9.7	8.9
Leucine	18.3	17.0	18.0	17.0
Lysine	16.4	18.0	19.0	18.0
Methionine	6.9	7.5	7.3	7.4
1-Methylhistidine^b^	ND	0.03	0.02	0.03
3-Methylhistidine^b^	ND	0.37	0.94	0.72
Phenylalanine	10.1	9.5	9.3	8.5
Threonine	8.5	8.6	8.6	7.9
Valine	13.0	13.0	13.0	12.0
Taurine^b^	ND	0.34	1.05	0.75
*Fatty acids*^b^ *(g/kg diet)*				
16:0	6.7	7.2	6.9	6.1
18:0	2.3	2.4	2.4	2.1
18:1 n-9	12.4	13.0	13.2	11.7
18:1 n-7	0.8	0.9	0.8	0.7
18:2 n-6	29.0	29.7	31.1	27.5
20:4 n-6	ND	0.04	ND	ND
18:3 n-3	3.4	3.5	3.7	3.3
20:5 n-3	ND	0.30	ND	ND
22:5 n-3	ND	0.03	ND	ND
22:6 n-3	ND	0.77	ND	ND

*BW-WM* blue whiting whole meal, *BW-HA* blue whiting protein hydrolysate prepared with Alcalase^®^, *BW-HP* blue whiting protein hydrolysate prepared with Protamex^®^, *ND* not detected

^a^Measured in diets after HCl-hydrolysis

^b^Only fatty acids found in concentrations > 0.5 g/kg diet, 20:4n-6 and long chain n-3 polyunsaturated fatty acids (20:5n-3, 22:5n-3, 22:6n-3) are shown

Body weight at baseline, body weight-to-body length ratio at euthanasia, weight of WATepi relative to body weight and daily energy and protein intake measured at week 4 were similar between groups (Table 4). The percent growth from baseline to end point was higher in the BW-HA and BW-HP groups compared to control group, and was higher in BW-HA group compared to BW-WM group. Otherwise, growth was similar between groups.

**Table 4:** Body weight, growth, body weight-to-body length ratio, WATepi weight, blood pressure, and dietary intake and urine output of sodium (Means and standard deviations)

	Control group	BW-WM group	BW-HA group	BW-HP group	ANOVA *p*
Body weight (g) at baseline	318 ± 8	325 ± 11	311 ± 7	323 ± 13	0.11
Growth (% change in body weight from baseline to end point)	72 ± 8^a^	76 ± 16^ab^	91 ± 11^c^	88 ± 4^bc^	0.030
Body weight-to-body length ratio (kg/m^2^) at end point	10.0 ± 0.6	10.1 ± 0.8	10.5 ± 0.5	10.8 ± 0.4	0.092
WATepi (g/kg BW)	29 ± 2	27 ± 3	31 ± 3	32 ± 4	0.12
Energy intake (kJ/kg BW/24 h)	863 ± 69	865 ± 183	943 ± 53	928 ± 66	0.48
Protein intake (g/kg BW/24 h)	9.4 ± 0.75	9.4 ± 1.99	10.4 ± 0.58	10.2 ± 0.72	0.40
Systolic blood pressure (mmHg) at baseline	114 ± 11	125 ± 9	123 ± 13	127 ± 15	0.31
Diastolic blood pressure (mmHg) at baseline	79 ± 11	89 ± 10	86 ± 11	88 ± 9	0.39
Sodium intake (mg/kg BW/24 h)	89 ± 7^a^	122 ± 26^b^	208 ± 12^c^	183 ± 13^d^	5.8 × 10− ^10^
Urine sodium (mmol/mmol creatinine)	21 ± 2^a^	23 ± 5^a^	29 ± 3^b^	34 ± 2^c^	1.3 × 10− ^5^

Data are presented as mean ± standard deviation for *N* = 5 rats in the control group, *N* = 6 rats in the BW-WM group, *N* = 6 rats in the BW-HA group and *N* = 6 rats in the BW-HP group. *p* values are shown for the comparisons of BW-WM group, BW-HA group, BW-HP group and control group using one-way ANOVA and the *p* values in the table show results from the one-way ANOVA comparisons. Fisher’s LSD was used as post hoc test when appropriate, and different letters indicate significant differences between groups

*BW-WM* blue whiting whole meal, *BW-HA* blue whiting protein hydrolysate prepared with Alcalase^®^, *BW-HP* blue whiting protein hydrolysate prepared with Protamex^®^, *WATepi* epididymal white adipose tissue, *ANOVA* analysis of variance, *LSD* least significant difference

*p* < 0.05 was considered significant

The sodium content was higher in all blue whiting protein diets compared to the control diet, resulting in a higher sodium intake in all blue whiting protein-fed groups compared to the control group, with the highest sodium intake in the BW-HA group (Table 4). Urine sodium concentration relative to creatinine was similar in the BW-WM group and control group and higher in both groups fed blue whiting protein hydrolysate, with the highest urine sodium concentration in the BW-HP group (Table 4). A strong positive correlation was observed between dietary sodium intake and urine sodium concentration (Pearson correlation 0.81 with two-tailed significance of *p* = 3.2 × 10^− 6^). Serum sodium concentration was within normal range for all rats (the mean for all rats was 140 with SD 1 mmol/l) with no differences between the groups (*p* ANOVA = 0.98, data not presented).

### Blood pressure

**Fig. 1:**
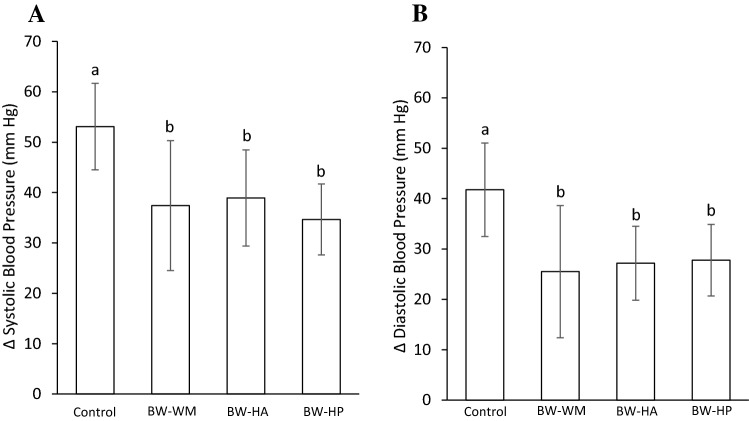
Increases in mean systolic blood pressure (**a)** and diastolic blood pressure (**b)** from baseline to end point. The figure shows values as the mean with their standard deviation shown by vertical bars for *N* = 5 rats in the control group, *N* = 6 rats in the BW-WM group, *N* = 6 rats in the BW-HA group and *N* = 6 rats in the BW-HP group. BW-WM group, BW-HA group, BW-HP group and control group were compared using one-way ANOVA. *p* ANOVA values were 0.031 and 0.047 for comparisons of systolic and diastolic blood pressure, respectively. Fisher’s LSD was used as post hoc test and different letters indicate significant differences between groups; *p* < 0.05 was considered significant; *BW-WM* blue whiting whole meal, *BW-HA* blue whiting protein hydrolysate prepared with Alcalase^®^, *BW-HP* blue whiting protein hydrolysate prepared with Protamex^®^, *ANOVA* analysis of variance, *LSD* least significant difference

Systolic and diastolic blood pressures were similar between groups at baseline (*p* ANOVA values were 0.31 and 0.39, respectively, Table 4). After 5 weeks intervention, the increases in both systolic and diastolic blood pressure were significantly smaller in the BW-WM group (*p* values 0.014 and 0.010, respectively), BW-HA group (*p* values 0.032 and 0.025, respectively) and BW-HP group (*p* values 0.0066 and 0.029, respectively) when compared to the control group (Figs. 1a, b). The increase from baseline to end point in systolic and diastolic blood pressures was similar between all groups fed blue whiting protein diets. The systolic and diastolic blood pressures were strongly correlated (Pearson correlation was 0.97 with two-tailed significance of *p* = 1.6 × 10^− 29^).

### Renin and ACE inhibition by dietary proteins

**Fig. 2:**
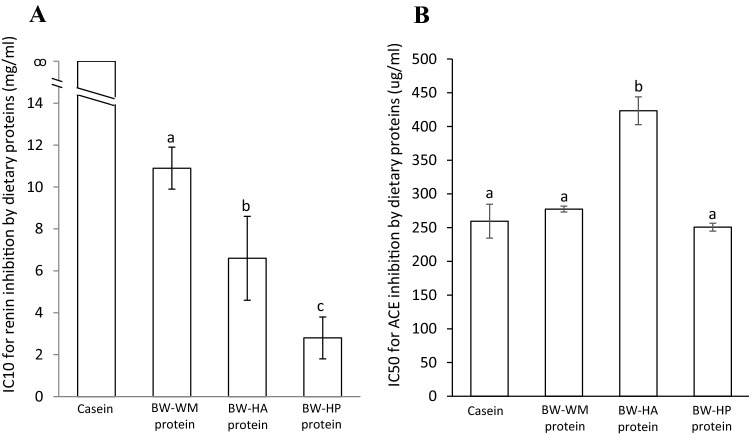
IC10 values for renin inhibition (**a**) and IC50 values for ACE inhibition (**b**) by dietary proteins. Data are presented as the amount of protein in ug/ml needed to inhibit 10% of renin activity and 50% of the ACE activity in a 0.25 U ACE assay, respectively. Data are presented as the mean with their standard error of mean shown by vertical bars for two different measurements. The dietary proteins were compared using one-way ANOVA. Casein was not included in the ANOVA analysis for renin inhibition since no measurable renin inhibition was detected for casein. Fisher’s LSD was used as post hoc test and different letters indicate significant differences between proteins; *p* < 0.05 was considered significant. Casein was prepared with trypsin; *BW-WM* blue whiting whole meal prepared with trypsin, *BW-HA* blue whiting protein hydrolysate prepared with Alcalase^®^, *BW-HP* blue whiting protein hydrolysate prepared with Protamex^®^, *ANOVA* analysis of variance, *LSD* least significant difference

For renin activity inhibition, no measurable inhibition was detected for casein, therefore casein was not included in the ANOVA analysis. The capacity for in vitro inhibition of renin activity was found to be most potent for BW-HP and slightly lower for BW-HA and BW-WM (Fig. 2a). The capacity for in vitro ACE inhibition was similar for casein, BW-WM protein and BW-HP protein, while the IC50 value for BW-HA protein was significantly higher (i.e., less potent) than the other dietary proteins including casein (Fig. 2b).

### Markers of kidney function, organ damage, inflammation and oxidative status

Serum concentrations of creatinine, cystatin C, alanine transaminase and aspartate transaminase were not significantly different between the experimental groups (Table 5). Also, no difference was seen between the groups for urine creatinine concentration. Urine concentrations (relative to creatinine) of total protein and cystatin C were abnormally high and comparable to concentrations previously seen in obese Zucker fa/fa rats with prominent hyperperfusion damage in podocytes [25, 40], but concentrations were similar in all groups (Table 5). Urine carbamide and uric acid concentrations were also abnormally high; however, the carbamide concentration was lower in all blue whiting protein-fed groups compared to the control group and uric acid concentration was lower in the BW-HA-fed rats, but similar in BW-WM and BW-HP-fed rats when compared to controls. The ammonium urine concentration was lower in the BW-HA and WW-HP groups when compared to the control and BW-WM groups. TIM-1 was found in kidneys from all rats in amounts ranging from 3 to 134 pg/mg protein, with no differences between the groups (data not presented, *p* ANOVA 0.57).

**Table 5:** Markers of kidney function, organ damage and inflammation (Means and standard deviations)

	Control group	BW-WM group	BW-HA group	BW-HP group	ANOVA *p*
Serum creatinine (µmol/l)	17.4 ± 1.1	17.0 ± 1.3	20.8 ± 4.0	18.0 ± 2.1	0.059
Serum cystatin C (ng/ml)	2266 ± 452	2382 ± 311	2565 ± 334	2451 ± 323	0.57
Serum alanine transaminase (U/l)	93 ± 37	98 ± 52	100 ± 21	86 ± 23	0.50
Serum aspartate transaminase (U/l)	143 ± 52	192 ± 134	198 ± 53	143 ± 47	0.48
Urine creatinine (mmol/l)	4.9 ± 1.0	3.9 ± 1.4	4.4 ± 1.3	4.8 ± 0.8	0.47
Urine total protein (g/mmol creatinine)	3.5 ± 1.7	3.8 ± 1.0	3.2 ± 1.4	3.8 ± 0.7	0.81
Urine cystatin C (µg/mmol creatinine)	1464 ± 891	1515 ± 175	1111 ± 323	1289 ± 298	0.55
Urine carbamide (mmol/mmol creatinine)	342 ± 23^a^	278 ± 36^b^	200 ± 15^c^	254 ± 15^b^	8.3 × 10− ^8^
Urine uric acid (µmol/mmol creatinine)	401 ± 41^a^	360 ± 51^ab^	293 ± 39^b^	329 ± 49^ab^	0.0066
Urine ammonium (µmol/mmol creatinine)	40.0 ± 16.7^a^	32.3 ± 9.0^a^	20.4 ± 2.4^b^	18.5 ± 2.3^b^	0.0051
Plasma total glutathione (µmol/L)	11 ± 2^a^	17 ± 6^b^	14 ± 2^ab^	14 ± 2^ab^	0.039
Plasma oxidized glutathione (µmol/L)	6 ± 1^a^	9 ± 3^b^	7 ± 1^ab^	8 ± 2^ab^	0.049
Plasma reduced glutathione (µmol/L)	5 ± 1	8 ± 3	7 ± 2	6 ± 2	0.33

Data are presented as mean ± standard deviation for *N* = 5 rats in the control group, *N* = 6 rats in the BW-WM group, *N* = 6 rats in the BW-HA group and *N* = 6 rats in the BW-HP group. *p* values are shown for the comparisons of BW-WM group, BW-HA group, BW-HP group and control group using one-way ANOVA and the *p* values in the table show results from the one-way ANOVA comparisons. Fisher’s LSD was used as post hoc test when appropriate, and different letters (a, b, c) indicate significant differences between groups

*BW-WM* blue whiting whole meal, *BW-HA* blue whiting protein hydrolysate prepared with Alcalase^®^, *BW-HP* blue whiting protein hydrolysate prepared with Protamex^®^, *WATepi* epididymal white adipose tissue, *ANOVA* analysis of variance, *LSD* least significant difference

*p* < 0.05 was considered significant

Serum concentrations of MCP-1, IL-1b, IL-6 and TNFα were similar between the experimental groups (data not presented). Plasma concentration of reduced glutathione was similar in all groups, whereas plasma concentrations of total and oxidized glutathione were significantly higher in the BW-WM group when compared to the control group, with no differences for either of these between the other groups (Table 5).

### n-3 and n-6 PUFAs in serum, liver and WATepi

The ratio of n-3/n-6 PUFA was higher in serum, liver and WATepi in the BW-WM group compared to the control group and both blue whiting hydrolysate groups (Supplemental Table). Rats fed BW-HA had lower ratio of n-3/n-6 ratio in serum when compared to the control group, otherwise no differences were seen in this ratio in serum, liver and WATepi for rats fed blue whiting hydrolysate diets compared to the control group. Rats fed BW-WM had higher levels of 20:5n-3 and 22:6n-3 in serum, higher levels of 20:5n-3 and 22:5n-3 in liver, and higher levels of 20:5n-3, 22:5n-3 and 22:6n-3 in WATepi. Serum level of 20:4n-6 was highest in BW-WM fed rats, but 20:4n-6 levels were not different from the other groups in liver and WATepi.

### Amino acids in plasma

Plasma concentration of α-aminobutyric acid was lower in rats fed BW-WM or BW-HA when compared to control group but was similar to the BW-HP group (Supplemental Table 2). Plasma glutamine concentration was lower in the BW-HA and BW-HP groups than in the control group, but was similar to the BW-WM group. The BW-WM fed rats had lower plasma methionine concentration compared to control rats and BW-HA-fed rats, but the concentration was similar to that in BW-HP-fed rats. Plasma taurine concentration was higher in BW-HA-fed rats compared to the control and BW-WM groups and similar to BW-HP-fed rats, with no difference between control rats and rats fed BW-WM or BW-HP diets. Plasma 3-methylhistidine concentration was higher in blue whiting protein-fed rats, in the order BW-HA > BW-HP > BW-WM > control groups. Otherwise, no differences were observed for plasma concentrations of amino acids and related compounds between the experimental groups.

### Amino acids in urine

Urine concentrations (relative to creatinine) of γ-aminobutyric acid and phenylalanine were lower in all blue whiting protein-fed groups, whereas 4-hydroxyproline concentration was higher when compared to the control group (Supplemental Table 3). Asparagine concentration was lower and glycine concentration was higher in urine in the BW-WM group compared to all other groups. Urine ornithine concentration was lower in the BW-HA and BW-HP groups compared to the control and BW-WM groups, whereas threonine concentration was lower in the BW-HA and BW-HP groups compared to the control, but was similar to that of the BW-WM group. Urine α-aminobutyric acid concentration was lower in the BW-HA group when compared to the other groups, and that of β-alanine was higher in the BW-HP group compared to BW-WM and BW-HA, but with no differences between the control group and blue whiting containing diets. The concentrations of 1-methylhistidine and 3-methylhistidine in urine were higher in rats fed BW-WM, BW-HA and BW-HP diets when compared to the control group. Urine 3-methylhistidine concentration was markedly higher in rats fed BW-HA and BW-HP diets compared to rats in the BW-WM group, whereas concentration of 1-methylhistidine was lower in rats fed BW-HA compared to the BW-WM and BW-HP groups. Urine taurine concentration was higher in the BW-HP group compared to BW-WM and BW-HA, but was similar to that of the control group. The total amount of free proteinogenic amino acids in urine was not different between the groups (*p* ANOVA = 0.20, data not presented).

## Discussion

In the present study, we show that diets containing 1/3 of total protein as blue whiting protein from either whole meal or protein hydrolysates attenuated the development of high blood pressure in obese Zucker fa/fa rats, but did not affect markers of kidney function, organ damage or inflammation. We chose to use obese Zucker fa/fa rats aged 8–9 weeks at the start of the intervention, since these rats develop an increase in blood pressure already before the age of 10 weeks [15–17] and spontaneously develop proteinuria and renal failure as they get older [18]. The obese Zucker fa/fa rats is considered to be a valuable experimental model for hypertension as it develops an age-related increase in blood pressure, as is also seen in humans [41].

The ability of the blue whiting proteins to attenuate the development of high blood pressure in obese Zucker fa/fa rats in the present study could be caused by the presence of bioactive peptides, e.g., peptides with physiological effects beyond being suppliers of amino acids, since antihypertensive peptides taken orally has been retrieved in its intact form in plasma in both rats and humans [42]. Blue whiting protein hydrolysates [23, 24] and Atlantic cod protein [19, 20, 43, 44] have been shown to inhibit ACE activity in vitro, and in addition Atlantic cod proteins are potent inhibitors of in vitro renin activity [19, 44]. Renin and ACE affect blood pressure by converting angiotensinogen via angiotensin I to the active vasoconstrictor angiotensin II in the circulation, and therefore inhibition of renin and/or ACE may lower blood pressure through reduced production of angiotensin II. All three blue whiting meals inhibited renin activity in vitro, whereas casein showed no measurable effect on renin inhibition. Renin is considered to be the rate-determining enzyme for production of angiotensin II [14], thus our findings from in vitro renin inhibition is in line with the lower blood pressure development in the blue whiting protein-fed rats. Compared to casein, ACE IC50 values of the blue whiting proteins were similar (BW-WM and BW-HP) or higher (BW-HA) and therefore did not correspond to the observed effects on blood pressure. Assessments of renin and ACE inhibitory activities of the protein hydrolysates with an in vitro assay are not sufficient for concluding whether the effects on blood pressure observed in vivo may or may not involve the ACE pathway. However, the findings that the stronger renin inhibitory in vitro activity of the blue whiting meals corresponds to the attenuated blood pressure increase in rats fed blue whiting diets are of interest and suggest that  the blood pressure regulation may be mediated through the renin–angiotensin system.

Proteinuria develops in the obese Zucker fa/fa rat already at around age 10 weeks [45] and decreased renal function is seen in obese Zucker fa/fa rats at around 12 weeks age [46], thus elevated urine concentrations (relative to creatinine) of total protein, free proteinogenic amino acids, cystatin C, carbamide and uric acid could be expected. Indeed, when compared to previous findings in male obese Zucker fa/fa rats [20, 25, 40], the urine concentrations of total protein, free proteinogenic amino acids, cystatin C, carbamide, uric acid and ammonium were abnormally high in all experimental groups with no difference between control group and blue whiting protein-fed groups, thus indicating renal dysfunction in all groups. Amino acids filtered by the glomeruli will normally be reabsorbed by the tubules and are therefore not excreted in urine [47], and the presence of proteins and amino acids in urine is amongst the earliest sign of almost all renal diseases in both humans and animals [48–50]. Serum creatinine is a commonly used marker of kidney function, since it is produced at a relatively constant rate mainly depending on the muscle mass, muscle function, diet and health status [51]. However, serum cystatin C [52] and urine cystatin C [53, 54] are considered to be better markers than serum creatinine for early detection of renal damage, in addition to the presence of proteins in urine [48]. Here, we found no differences between the groups for serum concentrations of creatinine and cystatin C. TIM-1 has emerged as a useful early indicator of tubular injury as TIM-1 is not detectable in normal kidney tissue, but is expressed on the proximal tubule apical membrane in response to renal injury [55]. In the present study, proteins were found in urine and TIM-1 was found in kidney homogenate to a similar extent in all rats, indicating that renal injury was evident in all groups. Since no differences were seen between the groups for serum creatinine concentrations, total urine concentration of proteinogenic amino acids, renal concentration of TIM-1 as well as concentrations of cystatin C in serum and urine, we conclude that dietary blue whiting proteins did not affect kidney function in these rats.

Plasma total and oxidized glutathione concentrations were higher in the BW-WM group compared to the control group, thus suggesting that the endogenous synthesis of the powerful antioxidant glutathione is upregulated, possibly to counteract and prevent lipid peroxidation in these hyperlipidemic rats [56]. Oxidative stress can potentially contribute to generation and maintenance of hypertension via inactivation of nitric oxide, which acts as vasodilator and regulates arterial tone [57, 58]. Glutathione plays an important role in nitric oxide metabolism by preventing the negative effects of nitric oxide scavenging by superoxide, and may thereby have an important function in blood pressure regulation [59]. Thus, the higher plasma concentration of glutathione in BW-WM-fed rats may partially explain the lower blood pressure increase in these rats when compared to the control group.

High fish intake [5–12] and fish oil supplementation [60] are associated with lower blood pressure. In the present study, the long-chain n-3 PUFAs 20:5n-3, 22:5n-3 and 22:6n-3 were found in the BW-WM diet, albeit in low amounts (0.11 wt%), but were not detected in the control diet or in the blue whiting protein hydrolysate containing diets. In line with this, the n-3/n-6 PUFA ratio was higher in serum, liver and white adipose tissue from BW-WM fed rats compared to both control and the blue whiting protein hydrolysate groups. A higher n-3/n-6 PUFA ratio could have contributed to the lower blood pressure increase in the BW-WM group by reducing the erythrocyte cell membrane arachidonic acid content, thereby suppressing the concentration of the contractile factor thromboxane A2 [61]. In contrast, the n-3/n-6 ratio in serum was lower in rats fed BW-HA diet compared to the control group, and otherwise no differences were seen between rats fed blue whiting hydrolysate containing diets and control group for n-3/n-6 ratio in serum, liver and WATepi. Since the blue whiting hydrolysate diets did not contain LC n-3 PUFA but still affected blood pressure development, it is not likely that the lower blood pressure increase in rats fed hydrolysed blue whiting proteins is mediated through n-3 PUFA and lower thromboxane A2 concentration. However, the higher n-3/n-6 PUFA may be a component in the delayed development of high blood pressure in rats fed the BW-WM diet.

Dietary factors other than peptides and long chain n-3 PUFAs, such as arginine, taurine and sodium, may have affected the blood pressure development in the present study. The higher arginine intake in the blue whiting protein-fed groups is of interest since arginine is a conditionally essential amino acid in rats [62] and serves as substrate for vascular production of the vasodilator nitric oxide [58]. Dietary supplementation of arginine has been shown to lower blood pressure in humans [63] and curb salt-induced blood pressure increase in salt-sensitive rats [64]. It has been suggested that dietary fish proteins attenuate development of hypertension due to higher arginine content in fish protein compared to casein [65]. Citrulline is a substrate for renal arginine synthesis [66], and increased citrulline accompanied by reduced arginine concentration in circulation has been shown in early stages of kidney disease [67]. The observation of no differences between the experimental groups for plasma and urine concentrations of citrulline and arginine further strengthens the assumption that blue whiting proteins did not affect kidney function in the present study. Still, the higher arginine intake may have contributed to the lower blood pressure increase in the blue whiting protein-fed groups.

Taurine supplementation has been shown to have antihypertensive properties in both humans and rats [68], and it has been shown that dietary taurine lower blood pressure in rats without affecting circulating or hepatic concentrations of taurine [69]. Taurine was found in all blue whiting protein containing diets, with the highest amounts in the hydrolysate diets; however, we observed no direct association between plasma taurine concentration and blood pressure development. The plasma elimination half-life for taurine after oral intake is estimated to be < 2 h in both rats and humans [70, 71], and since our rats were fasted for 12 h before blood sampling, the lack of association between dietary taurine intake and plasma concentration of taurine can most likely be explained by the rapid turnover of taurine. The higher dietary intake of taurine in rats fed blue whiting protein diets may be among the nutrients contributing to the lower blood pressure increase in these groups.

High sodium intake can increase blood pressure, as sodium acts as a vasoconstrictor and controls blood volume by increasing arterial constriction and peripheral vascular resistance [72]. Despite the higher sodium intake in blue whiting protein-fed rats when compared to the control rats, the blood pressure increase was lower in blue whiting protein-fed rats and circulating sodium concentrations were similar in all groups. The strong positive correlation between dietary sodium intake and urine sodium excretion indicates that the kidneys in rats fed blue whiting protein diets coped well with the higher sodium dietary load by excreting sodium in the urine and maintaining serum sodium concentration within normal range. However, we cannot exclude the possibility that the blue whiting proteins might have had more pronounced effect on the blood pressure development in the rats if the sodium content was lower in the diets, especially in the BW-HA and BW-HP groups.

Diets containing the blue whiting proteins seem to have little effects on the concentrations of proteinogenic amino acids in plasma and urine when compared to the control diet, whereas concentrations of non-proteinogenic amino acids differed to a larger extent between the groups. We have recently shown that serum and urine concentrations of 1-methylhistidine were increased after 8 weeks with a weekly intake of 750 g of Atlantic cod fillet in healthy adults with overweight/obesity, whereas serum and urine concentrations of 3-methylhistidine were not affected [73]. When obese Zucker fa/fa rats were fed diets containing Atlantic cod fillet proteins (25% of total protein intake), we found higher concentrations of 1-methylhistidine and 3-methylhistidine in both plasma and urine when compared to rats fed diets containing milk proteins as the sole protein source [20]. In the present study, 3-methylhistidine and β-alanine were detected only in diets containing blue whiting protein meals after HCl-hydrolysis, with the highest levels in BW-HA and BW-HP, whereas 1-methylhystidine was found in comparable amounts in the three blue whiting diets and not in the control diet. 1- and 3-methylhistidine are also found in rat muscle in anserine (a dipeptide of β-alanine and 1-methylhistidine) and carnosine (a dipeptide of β-alanine and histidine), and in skeletal and intestinal muscle proteins [74, 75]. Although elevated plasma and urine concentration of 3-MeHis could indicate increased proteolysis, this is valid only when the diet is devoid of 3-MeHis. These methylhistidines are not reutilized for protein synthesis or metabolized but are excreted in the urine, and in line with the dietary contents the urine concentrations of 1- and 3-methylhistidine were higher in rats fed diets containing blue whiting protein meal, especially the hydrolysed forms. β-Alanine, on the other hand, can be metabolized to CO_2_, malonyl-CoA or acetyl-CoA, thus explaining the smaller differences in urine β-alanine concentrations between the dietary groups when compared to the concentrations of the methylhistidines. Thus, the higher urine and plasma concentrations of 3-methylhistidine in rats fed diets containing blue whiting proteins are most likely reflections of the dietary intake and not of muscle protein catabolism.

4-Hydroxyproline was found only in diets containing blue whiting proteins, and rats fed these diets had higher urine 4-hydroxyproline concentration compared to the control group. 4-Hydroxyproline is excreted mainly by the lungs (about 75%) as CO_2_ and by the kidneys (25%) primarily as proline–hydroxyproline and glycine–proline–hydroxyproline due to low peptidase activity [76]. Thus, the higher urine 4-hydroxyproline in blue whiting protein-fed rats probably does not indicate a larger degradation of collagen in connective tissue in these rats, but is most likely a reflection of the 4-hydroxyproline intake.

The present study has some methodological strengths and limitations. Dietary sodium content was higher in all blue whiting protein diets compared to control, and although rats seemed to efficiently excrete excess sodium in urine, it is possible that the effects on blood pressure in the blue whiting protein-fed groups would have been more prominent if dietary sodium intake was lower. Blood pressure was measured using the tail-cuff method (volume-pressure recording) at baseline and near end point of the intervention period. The tail-cuff method is a non-invasive and inexpensive method that does not require surgery, and was chosen instead of continuous intravascular blood pressure measured by telemetry, since comparison of these methods shows similar results over the physiological range of blood pressure in mice [77]. In future studies, we should consider extending the experiment until the rats reach a higher blood pressure, since we have observed in our laboratory that rats from the same breeder as used in the present study reached a blood pressure that was 15–20 mmHg higher than at end point in the present study. Also, blood pressure should be measured weekly to better monitor blood pressure development. A strength of the present study is that we used obese Zucker fa/fa rat, which is a valuable experimental model for hypertension as it develops an age-related increase in blood pressure, as is also seen in humans [41]. This study was designed to investigate the effects of intact and hydrolysed blue whiting meals in diets with 1/3 of protein from blue whiting meal on the development of high blood pressure using an experimental design that is relevant to human nutrition. To reduce the number of animals, and in line with the 3Rs, we did not include lean Zucker rats since they do not experience an increase in blood pressure as they age, at least not in the age span relevant to the present study. Assessments of renin and ACE inhibitory activities of the protein hydrolysates with an in vitro assay are not sufficient for concluding whether the lower blood pressure increase observed in vivo mediated through the ACE pathway, and in vivo analyses of ACE activity and renin activity should be implemented in future studies with similar design. The high sodium content in the fish protein hydrolysates may have affected the outcome of the study, and it is possible that the effects on blood pressure could have been even more prominent if sodium in the fish meals had been removed. Future studies with fish protein hydrolysates should aspire to remove excess sodium and at the same time retain short peptides and free amino acids, e.g., by using a reverse osmosis filter. The present study is small, but will constitute a base for sample size calculations for future studies with similar designs.

To conclude, the findings in this study demonstrate that proteins from blue whiting may attenuate the development of high blood pressure in obese Zucker fa/fa rats, and the effect was similar for intact and enzymatically hydrolysed blue whiting proteins. Our in vitro studies suggest that the effects of blue whiting protein on blood pressure development may be mediated through the renin–angiotensin system, by inhibition of renin activity but not through inhibition of ACE. The blue whiting proteins did not affect markers of kidney function when compared to the control group, and all groups showed signs of poor renal function with elevated urine concentrations of proteins and cystatin C. The findings in the present study suggest that blue whiting proteins may have a potential as functional food ingredients in the dietary prevention of high blood pressure in obesity.

## Electronic supplementary material

Below is the link to the electronic supplementary material.

Supplementary file1 (DOCX 33 kb)
