# Supplementary material for: Effects of intact and hydrolysed blue whiting proteins on blood pressure and markers of kidney function in obese Zucker fa/fa rats
Source: Eur J Nutr. 2020 May 14;60(1):529–44. doi: 10.1007/s00394-020-02262-9 (PMC7867508; doi:10.1007/s00394-020-02262-9)
Supplement: Supplementary file 1 — Supplementary file1 (DOCX 33 kb) [file 394_2020_2262_MOESM1_ESM.docx]

**Supplemental Table 1**: n-3 and n-6 PUFAs in serum, liver and epididymal white adipose tissue.

(Means and standard deviations)

| g/100g | Control group | BW-WM group | BW-HA group | BW-HP group | ANOVA p |
| --- | --- | --- | --- | --- | --- |
| Serum | | | | | |
| 18:3 n-3 | 0.49 ± 0.22 | 0.61 ± 0.08 | 0.42 ± 0.09 | 0.52 ± 0.17 | 0.21 |
| 20:5 n-3 | 0.49 ± 0.13^a^ | 1.02 ± 0.14^b^ | 0.59 ± 0.17^a^ | 0.66 ± 0.13^a^ | 3.1x10^-5^ |
| 22:5 n-3 | 0.80 ± 0.12 | 0.88 ± 0.11 | 0.70 ± 0.14 | 0.78 ± 0.11 | 0.10 |
| 22:6 n-3 | 3.28 ± 0.26^a^ | 4.54 ± 0.28^b^ | 2.92 ± 0.14^c^ | 3.00 ± 0.29^ac^ | 1.4x10^-9^ |
| Sum n-3 PUFA | 5.05 ± 0.51^a^ | 7.06 ± 0.36^b^ | 4.63 ± 0.35^a^ | 4.96 ± 0.41^a^ | 9.7x10^-9^ |
| 18:2 n-6 | 9.50 ± 1.48^a^ | 13.73 ± 1.21^b^ | 12.07 ± 1.38^bc^ | 11.72 ± 1.75^c^ | 0.0015 |
| 18:3 n-6 | 0.31 ± 0.04^a^ | 0.31 ± 0.04^a^ | 0.42 ± 0.09^b^ | 0.42 ± 0.06^b^ | 0.012 |
| 20:3 n-6 | 0.66 ± 0.08^a^ | 1.43 ± 0.41^b^ | 0.85 ± 0.21^a^ | 0.91 ± 0.15^a^ | 3.9x10^-4^ |
| 20:4 n-6 | 29.6 ± 4.7^a^ | 20.8 ± 1.9^b^ | 30.8 ± 2.7^a^ | 28.7 ± 3.4^a^ | 1.6x10^-4^ |
| 22:4 n-6 | 0.76 ± 0.06^a^ | 0.41 ± 0.10^b^ | 0.56 ± 0.06^c^ | 0.63 ± 0.07^c^ | 2.9x10^-6^ |
| 22:5 n-6 | 0.47 ± 0.02^a^ | 0.20 ± 0.04^b^ | 0.51 ± 0.08^a^ | 0.50 ± 0.05^a^ | 3.6x10^-9^ |
| Sum n-6 PUFA | 41.3 ± 3.31^a^ | 36.9 ± 1.6^b^ | 45.2 ± 3.2^c^ | 42.9 ± 2.5^ac^ | 3.5x10^-4^ |
| Ratio n-3/n-6 PUFA | 0.123 ± 0.020^a^ | 0.192 ± 0.012^b^ | 0.103 ± 0.013^c^ | 0.116 ± 0.038^ac^ | 1.1x10^-8^ |
| Liver | | | | | |
| 18:3 n-3 | 0.52 ± 0.10^a^ | 0.82 ± 0.27^b^ | 0.42 ± 0.09^a^ | 0.50 ± 0.10^a^ | 0.0017 |
| 20:5 n-3 | 0.10 ± 0.05^a^ | 0.25 ± 0.13^b^ | 0.07 ± 0.02^a^ | 0.11 ± 0.03^a^ | 0.0037 |
| 22:5 n-3 | 0.28 ± 0.09^a^ | 0.43 ± 0.20^b^ | 0.22 ± 0.06^a^ | 0.26 ± 0.06^a^ | 0.031 |
| 22:6 n-3 | 1.56 ± 0.77^ab^ | 1.92 ± 0.69^a^ | 0.92 ± 0.25^b^ | 1.25 ± 0.33^b^ | 0.030 |
| sum n-3 PUFA | 2.46 ± 0.99^a^ | 3.42 ± 1.2^ab^ | 1.63 ± 0.31^b^ | 2.11 ± 0.49^b^ | 0.0094 |
| 18:2 n-6 | 8.80 ± 0.92^a^ | 10.74 ± 2.14^b^ | 7.77 ± 1.01^a^ | 8.24 ± 0.74^a^ | 0.0061 |
| 18:3 n-6 | 0.21 ± 0.02 | 0.19 ± 0.03 | 0.22 ± 0.04 | 0.22 ± 0.04 | 0.47 |
| 20:3 n-6 | 0.22 ± 0.11^a^ | 0.46 ± 0.18^b^ | 0.19 ± 0.05^a^ | 0.26 ± 0.09^a^ | 0.0045 |
| 20:4 n-6 | 4.80 ± 2.23 | 2.96 ± 1.27 | 3.12 ± 0.82 | 4.13 ± 1.10 | 0.13 |
| 22:4 n-6 | 0.18 ± 0.05 | 0.15 ± 0.06 | 0.13 ± 0.03 | 0.16 ± 0.03 | 0.37 |
| 22:5 n-6 | 0.16 ± 0.06^a^ | 0.08 ± 0.04^b^ | 0.14 ± 0.04^a^ | 0.17 ± 0.03^a^ | 0.010 |
| Sum n-6 PUFA | 14.4 ± 3.2 | 14.6 ± 3.1 | 11.6 ± 1.5 | 13.2 ± 1.7 | 0.17 |
| Ratio n-3/n-6 PUFA | 0.166 ± 0.028^a^ | 0.229 ± 0.033^b^ | 0.140 ± 0.011^a^ | 0.159 ± 0.017^a^ | 2.2x10^-5^ |
| Epididymal white adipose tissue | | | | |  |
| 18:3 n-3 | 1.58 ± 0.21 | 1.73 ± 0.09 | 1.55 ± 0.11 | 1.64 ± 0.14 | 0.16 |
| 20:5 n-3 | 0.03 ± 0.01^a^ | 0.07 ± 0.01^b^ | 0.03 ± 0.01^a^ | 0.03 ± 0.01^a^ | 3.0x10^-9^ |
| 22:5 n-3 | 0.14 ± 0.01^a^ | 0.19 ± 0.02^b^ | 0.13 ± 0.01^a^ | 0.13 ± 0.02^a^ | 1.5x10^-5^ |
| 22:6 n-3 | 0.15 ± 0.03^a^ | 0.36 ± 0.05^b^ | 0.10 ± 0.01^c^ | 0.12 ± 0.03^ac^ | 3.7x10^-11^ |
| Sum n-3 PUFA | 1.90 ± 0.25^a^ | 2.35 ± 0.13^b^ | 1.81 ± 0.11^a^ | 1.92 ± 0.18^a^ | 1.4x10^-4^ |
| 18:2 n-6 | 16.8 ± 2.0 | 18.2 ± 1.4 | 1.1 ± 0.9 | 17.1 ± 1.2 | 0.096 |
| 18:3 n-6 | 0.07 ± 0.01 | 0.07 ± 0.01 | 0.07 ± 0.01 | 0.07 ± 0.02 | 0.61 |
| 20:3 n-6 | 0.18 ± 0.02^a^ | 0.20 ± 0.02^b^ | 0.15 ± 0.01^a^ | 0.17 ± 0.02^a^ | 0.0024 |
| 20:4 n-6 | 0.46 ± 0.05 | 0.37 ± 0.05 | 0.40 ± 0.07 | 0.40 ± 0.07 | 0.12 |
| 22:4 n-6 | 0.16 ± 0.02^a^ | 0.11 ± 0.02^b^ | 0.14 ± 0.02^ac^ | 0.13 ± 0.02^bc^ | 0.0085 |
| 22:5 n-6 | 0.06 ± 0.01^a^ | 0.04 ± 0.01^b^ | 0.05 ± 0.01^ab^ | 0.05 ± 0.01^ab^ | 0.023 |
| sum n-6 PUFA | 17.7 ± 2.1 | 19.0 ± 1.5 | 16.9 ± 1.0 | 17.9 ± 1.3 | 0.13 |
| Ratio n-3/n-6 PUFA | 0.107 ± 0.003^a^ | 0.124 ± 0.009^b^ | 0.107 ± 0.002^a^ | 0.107 ± 0.005^a^ | 3.6x10^-5^ |

The table shows values as the mean with their standard deviation for N = 5 rats in the control group, N = 6 rats in the BW-WM group, N = 6 rats in the BW-HA group and N = 6 rats in the BW-HP group. One-Way ANOVA with p < 0.05 was followed by Fischer’s LSD to determine significant differences between groups. Different letters indicate significant differences between groups. p < 0.05 was considered significant. BW-WM: blue whiting whole meal; BW-HA: blue whiting protein hydrolysate prepared with Alcalase®; BW-HP: blue whiting protein hydrolysate prepared with Protamex®; ANOVA: analysis of variance; LSD: least significant difference

**Supplemental Table 2:** Amino acids in plasma

(Means and standard deviations)

| µmol/l | Control group | BW-WM group | BW-HA group | BW-HP group | ANOVA  P |
| --- | --- | --- | --- | --- | --- |
| Alanine | 304 ± 25 | 332 ± 57 | 316 ± 35 | 317 ± 34 | 0.72 |
| α-aminobutyric acid | 66.8 ± 6.0^a^ | 42.6 ± 6.9^b^ | 41.7 ± 20.8^b^ | 52.0 ± 9.6^ab^ | 0.014 |
| Arginine | 70.2 ± 17.4 | 60.7 ± 31.0 | 75.8 ± 24.1 | 55.5 ± 21.8 | 0.49 |
| Asparagine | 45.8 ± 3.3 | 43.9 ± 3.4 | 46.0 ± 4.4 | 44.3 ± 4.5 | 0.75 |
| Aspartic acid | 8.82 ± 1.10 | 9.89 ± 2.67 | 7.43 ± 1.74 | 7.80 ± 1.88 | 0.17 |
| Citrulline | 55.7 ± 4.1 | 63.1 ± 9.0 | 55.3 ± 12.8 | 55.3 ± 8.3 | 0.41 |
| Cystine | 23.2 ± 5.5 | 29.0 ± 6.8 | 29.2 ± 4.4 | 27.8 ± 3.7 | 0.25 |
| Glutamic acid | 98.1 ± 22.6 | 104.8 ± 13.0 | 85.0 ± 21.2 | 83.5 ± 20.3 | 0.21 |
| Glutamine | 457 ± 33^a^ | 417 ± 47^ab^ | 381 ± 70^b^ | 369 ± 20^b^ | 0.025 |
| Glycine | 123 ± 13 | 106 ± 11 | 108 ± 17 | 103 ± 12 | 0.093 |
| Histidine | 51.5 ± 4.2 | 49.7 ± 3.9 | 52.9 ± 5.1 | 50.1 ± 4.0 | 0.58 |
| 4-Hydroxyproline | 20.1 ± 0.45 | 20.0 ± 1.7 | 19.2 ± 1.5 | 18.5 ± 2.2 | 0.34 |
| Isoleucine | 88.0 ± 8.0 | 92.9 ± 7.9 | 98.2 ± 7.9 | 89.3 ± 8.7 | 0.30 |
| Leucine | 138 ± 12 | 145 ± 20 | 155 ± 14 | 141 ± 14 | 0.32 |
| Lysine | 547 ± 108 | 583 ± 220 | 581 ± 117 | 441 ± 127 | 0.35 |
| Methionine | 42.7 ± 0.5^ab^ | 37.0 ± 2.5^c^ | 43.1 ± 3.0^a^ | 39.7 ± 3.9^bc^ | 0.0049 |
| 1-Methylhistidine | 5.06 ± 2.79 | 4.81 ± 0.64 | 4.36 ± 0.87 | 4.16 ± 0.83 | 0.72 |
| 3-Methylhistidine | 6.43 ± 0.45^a^ | 14.4 ± 2.3^b^ | 36.1 ± 6.1^c^ | 24.5 ± 6.5^d^ | 1.6x10^-8^ |
| O-phosphoethanolamine | 7.24 ± 0.41 | 7.61 ± 0.51 | 7.80 ± 0.98 | 7.57 ± 0.88 | 0.68 |
| O-phosphoserine | 12.3 ± 1.0 | 14.1 ± 4.1 | 13.1 ± 1.8 | 13.3 ± 1.8 | 0.70 |
| Ornithine | 58.0 ± 7.3 | 81.2 ± 28.3 | 64.1 ± 14.3 | 67.6 ± 11.3 | 0.19 |
| Phenylalanine | 63.9 ± 6.5 | 62.3 ± 3.3 | 62.7 ± 2.8 | 63.1 ± 4.5 | 0.94 |
| Proline | 89.2 ± 3.8 | 89.5 ± 9.4 | 103.0 ± 28.3 | 92.4 ± 14.9 | 0.49 |
| Serine | 184 ± 6 | 171 ± 9 | 171 ± 24 | 180 ± 23 | 0.53 |
| Taurine | 264 ± 19^a^ | 272 ± 27^a^ | 318 ± 36^b^ | 287 ± 22^ab^ | 0.017 |
| Threonine | 163 ± 20 | 136 ± 20 | 155 ± 21 | 168 ± 33 | 0.15 |
| Tryptophan | 56.4 ± 6.4 | 57.7 ± 5.2 | 49.3 ± 11.9 | 52.6 ± 4.4 | 0.26 |
| Tyrosine | 74.2 ± 6.9 | 69.3 ± 7.2 | 79.8 ± 8.8 | 71.0 ± 10.6 | 0.20 |
| Valine | 148 ± 8 | 160 ± 28 | 175 ± 24 | 158 ± 25 | 0.33 |

Data are presented as mean ± standard deviation for N = 5 rats in the control group, N = 6 rats in the BW-WM group, N = 6 rats in the BW-HA group and N = 6 rats in the BW-HP group. P values are shown for the comparisons of BW-WM group, BW-HA group, BW-HP group and control group using one-way ANOVA and the P values in the table show results from the one-way ANOVA comparisons. Fischer’s LSD was used as post hoc test when appropriate, and different letters indicate significant differences between groups; p < 0.05 was considered significant; BW-WM: blue whiting whole meal; BW-HA: blue whiting protein hydrolysate prepared with Alcalase®; BW-HP: blue whiting protein hydrolysate prepared with Protamex®; WATepi: epididymal white adipose tissue; ANOVA: analysis of variance; LSD: least significant difference.

**Supplemental Table 3:** Amino acids in urine

(Means and standard deviations)

| µmol/mmol creatinine | Control group | BW-WM group | BW-HA group | BW-HP group | ANOVA  P |
| --- | --- | --- | --- | --- | --- |
| Alanine | 37.5 ± 5.3 | 42.9 ± 8.0 | 35.3 ± 9.8 | 39.2 ± 7.9 | 0.43 |
| α-aminobutyric acid | 1.22 ± 0.24^a^ | 1.29 ± 0.31^a^ | 0.73 ± 0.16^b^ | 1.29 ± 0.45^a^ | 0.015 |
| Arginine | 12.4 ± 2.3 | 13.4 ± 2.4 | 11.0 ± 2.6 | 11.7 ± 2.5 | 0.41 |
| Asparagine | 8.27 ± 1.20^a^ | 4.09 ± 2.34^b^ | 6.24 ± 1.71^a^ | 6.38 ± 1.54^a^ | 0.0091 |
| Aspartic acid | 5.27 ± 0.94 | 4.86 ± 1.91 | 4.61 ± 2.24 | 4.00 ± 0.96 | 0.68 |
| β-alanine | 36.9 ± 2.1^ab^ | 34.1 ± 3.2^a^ | 32.1 ± 4.6^a^ | 40.0 ± 6.0^b^ | 0.028 |
| Citrulline | 2.37 ± 0.26 | 2.21 ± 0.60 | 2.20 ± 0.50 | 2.14 ± 0.32 | 0.87 |
| γ-aminobutyric acid | 8.59 ± 0.85^a^ | 6.21 ± 0.75^b^ | 4.64 ± 0.44^c^ | 5.02 ± 0.38^c^ | 1.1x10^-8^ |
| Cystine | 22.2 ± 5.0 | 28.8 ± 9.5 | 21.4 ± 6.3 | 22.4 ± 7.7 | 0.32 |
| Glutamic acid | 14.7 ± 2.5 | 21.8 ± 9.6 | 18.3 ± 4.1 | 16.7 ± 3.5 | 0.23 |
| Glutamine | 35.2 ± 5.8 | 30.9 ± 11.3 | 35.0 ± 11.9 | 33.1 ± 6.6 | 0.86 |
| Glycine | 37.7 ± 6.1^a^ | 49.1 ± 9.7^b^ | 35.4 ± 7.1^a^ | 35.0 ± 4.0^a^ | 0.0085 |
| Histidine | 13.7 ± 6.8 | 14.8 ± 3.3 | 12.2 ± 3.0 | 12.5 ± 3.9 | 0.66 |
| 4-Hydroxyproline | 0.90 ± 0.21^a^ | 2.26 ± 1.16^b^ | 1.94 ± 1.03^b^ | 1.87 ± 0.68^b^ | 0.0026 |
| Isoleucine | 12.8 ± 1.5 | 13.0 ± 3.7 | 9.5 ± 4.3 | 14.1 ± 1.7 | 0.98 |
| Leucine | 17.5 ± 4.2 | 17.6 ± 6.7 | 17.6 ± 8.8 | 24.9 ± 7.0 | 0.22 |
| Lysine | 88.7 ± 19.1 | 91.8 ± 25.3 | 55.1 ± 32.9 | 71.8 ± 41.9 | 0.23 |
| Methionine | 18.3 ± 4.3 | 20.5 ± 10.3 | 16.0 ± 10.0 | 13.3 ± 4.4 | 0.46 |
| 1-Methylhistidine | 8.15 ± 1.23^a^ | 32.8 ± 6.9^b^ | 24.5 ± 5.6^c^ | 34.7 ± 5.2^b^ | 5.1x10^-7^ |
| 3-Methylhistidine | 4.03 ± 1.13^a^ | 47.0 ± 17.1^b^ | 214.6 ± 76.4^c^ | 163.1 ± 45.8^c^ | 2.1x10^-6^ |
| O-phosphoethanolamine | 16.0 ± 3.3 | 25.9 ± 12.4 | 18.5 ± 5.2 | 17.7 ± 4.0 | 0.14 |
| O-phosphoserine | 43.8 ± 5.5 | 49.8 ± 12.3 | 40.7 ± 5.4 | 42.6 ± 3.6 | 0.22 |
| Ornithine | 4.10 ± 0.56^a^ | 5.23 ± 1.66^a^ | 3.27 ± 0.99^b^ | 3.26 ± 0.93^b^ | 0.021 |
| Phenylalanine | 22.3 ± 3.6^a^ | 17.1 ± 2.4^b^ | 14.9 ± 3.3^b^ | 17.1 ± 2.8^b^ | 0.0058 |
| Proline | 24.6 ± 6.8 | 26.8 ± 6.5 | 19.5 ± 5.9 | 18.8 ± 3.7 | 0.077 |
| Serine | 19.6 ± 2.9 | 12.9 ± 7.4 | 10.5 ± 4.6 | 14.6 ± 4.7 | 0.061 |
| Taurine | 4896 ± 618^ab^ | 4602 ± 400^a^ | 4271 ± 182^a^ | 5236 ± 681^b^ | 0.037 |
| Threonine | 44.6 ± 6.2^a^ | 35.3 ± 4.4^ab^ | 27.7 ± 7.0^b^ | 29.2 ± 10.6^b^ | 0.0062 |
| Tyrosine | 11.6 ± 3.1 | 12.4 ± 5.7 | 8.6 ± 3.8 | 9.4 ± 2.0 | 0.32 |
| Valine | 15.8 ± 3.6 | 17.5 ± 4.4 | 13.7 ± 3.4 | 14.5 ± 2.5 | 0.30 |

Data are presented as mean ± standard deviation for N = 5 rats in the control group, N = 6 rats in the BW-WM group, N = 6 rats in the BW-HA group and N = 6 rats in the BW-HP group. P values are shown for the comparisons of BW-WM group, BW-HA group, BW-HP group and control group using one-way ANOVA and the P values in the table show results from the one-way ANOVA comparisons. Fischer’s LSD was used as post hoc test when appropriate, and different letters indicate significant differences between groups; p < 0.05 was considered significant; BW-WM: blue whiting whole meal; BW-HA: blue whiting protein hydrolysate prepared with Alcalase®; BW-HP: blue whiting protein hydrolysate prepared with Protamex®; WATepi: epididymal white adipose tissue; ANOVA: analysis of variance; LSD: least significant difference.
